# The current knowledge gap on metallothionein mediated metal-detoxification in Elasmobranchs

**DOI:** 10.7717/peerj.10293

**Published:** 2020-11-02

**Authors:** Rachel Ann Hauser-Davis

**Affiliations:** Laboratório de Avaliação e Promoção a Saúde Ambiental, Instituto Oswaldo Cruz, Oswaldo Cruz Foundation, Rio de Janeiro, Brazil

**Keywords:** Metallothionein, Metal contamination, Detoxification, Pollution, Sharks, Rays and skates

## Abstract

Elasmobranchs are particularly vulnerable to environmental contamination, especially pollutants that may bioaccumulate and biomagnify, throughout the trophic web, such as metals. However, Elasmobranch management and conservation plans are challenging, and this group is often neglected regarding ecotoxicological analyses, particularly concerning metal detoxification mechanisms. This article discusses metallothionein (MT) mediated metal detoxification in Elasmobranchs and reflects on the current knowledge gap in this regard.

## Introduction

Approximately 30% of all elasmobranchs worldwide are threatened with extinction as defined by the International Union for Conservation of Nature (IUCN) (Dulvy et al., 2014) and, 47% are classified as data deficient, indicating absence of minimal information to conduct any type of evaluation (Mace et al., 2008; Dulvy et al., 2014). The International Plan of Action for the Conservation and Management of Sharks (IPOA-Sharks), which aims to ensure the conservation and management of shark stocks and their long-term sustainable use, has defined several important issues regarding the conservation of Elasmobranchs, including the need for biodiversity maintenance through shark population viability, the management requirements of shark fishery resources for sustainable use and the need for habitat protection (FAO, 2020). Specifically concerning habitat protection, the IPOA-Sharks indicates that several anthropogenic activities (i.e., fishing, aquaculture, ecotourism, dredging, mining, catchment area clearing, dumping, nutrient enrichment, pollution and the introduction of exotic organisms) are the cause of broad-scale habitat and/or loss of critical habitats, such as nursery, pupping and mating areas or migration lanes. In this regard, elasmobranchs are particularly vulnerable to environmental chemical contamination, due to their high lipid content, long life spans, high trophic level, delayed maturation, long gestation, and small litters (Stevens et al., 2000; Van der Oost, Beyer & Vermeulen, 2003; Endo et al., 2008; Dulvy et al., 2014). Furthermore, the use of coastal near-shore areas for breeding and/or as nursery grounds by many species may lead to increased pollutant exposure (Gelsleichter & Walker, 2010), as about 2.4 billion people, 40% of the world’s population, live within 100 km of coastlines. This, in turn, has increased population density and economic activities, furthering pressures, that is, environmental contamination and habitat degradation, on coastal ecosystems (Fleming & Laws, 2006).

Ecotoxicological analyses are valuable tools in this regard, as sublethal pollutant biochemical outcome information may be obtained. This allows for decision-making on the mitigation of harmful pollutant effects, as regulatory decisions can be partly based on data from peer-reviewed literature, with wide-reaching implications for environmental protection (Hanson et al., 2017). However, Elasmobranch management and conservation plans are challenging (Shotton, 1999; Dulvy et al., 2017) and this group is often neglected regarding ecotoxicological analyses, particularly concerning metal detoxification mechanisms, indicating that further assessments are required (WWF, 2019).

Most Elasmobranch pollutant exposure assessments have focused on muscle tissue contaminant loads in the context of growing concerns regarding dietary human exposure (Squadrone et al., 2015; Boldrocchi et al., 2019), instead of contaminant effects on organism health. In this regard, data on teleosts are more readily available than in Elasmobranchs, and some toxic physiological thresholds set for teleosts, that is, concerning morphological abnormalities, altered behaviour, histopathological effects and reproductive alterations (Webber & Haines, 2003; Adams et al., 2010; Sandheinrich & Wiener, 2011) have been reported as significantly exceeded in shark tissues (Adams et al., 2010). Elasmobranch health, in fact, is rarely taken into account in these assessments, although studies concerning oxidative stress, homeostatic balance and, more recently, some health indicators and reduced ability to deal with stressors have been published (De Boeck, Grosell & Wood, 2001; Kinne-Saffran & Kinne, 2001; Eyckmans et al., 2013; Alves et al., 2016; Lyons & Wynne-Edwards, 2019; Merly et al., 2019).

Among the diverse environmental contaminants present in aquatic ecosystems, the ones of greatest concern are those that exhibit environmental persistence, bioavailability, the capability of bioaccumulating throughout in the trophic chain and toxic effects, like metals (Singh et al., 2011). These contaminants can be found in aquatic organism tissues at several orders of magnitude above concentrations identified in the water column (Jabeen & Chaudhry, 2010). Therefore, the determination of metals and metalloids in tissues and organs is an important method to assess contamination by these pollutants in aquatic ecosystems (Van Den Heuvel, 2004). However, most studies in this context are available only regarding full metal loads and do not take into account subcellular bioavailability. These contaminants, when entering the cell, can undergo compartmentalization, affecting their bioavailability (Wallace & Luoma, 2003; Marijić & Raspor, 2006) and, consequently, their potential to cause deleterious cellular effects, demonstrating that total metal assessments are not adequate to indicate the harmful effects of environmental contamination (Decataldo et al., 2004). This is more adequately evaluated through the use of biomarkers.

Biomarkers express measurable biological changes that indicate exposure to certain pollutants, allowing for the determination of sublethal pollutant effects which arise prior to severe deleterious effects and organism death (Hagger et al., 2006). This type of early pollutant impact assessment allows not only for the protection of wild species in a biodiversity conservation context, but also for environment health assessments (Van der Oost, Beyer & Vermeulen, 2003), decision-making and direct human risk mitigation actions.

Certain proteins exhibiting the ability to bind to metals, termed metalloproteins, are considered potential biomarkers of exposure to metals, and can be applied in the biomonitoring of environmental impacts and in the evaluation of the efficiency of the procedures adopted to reduce these impacts (Lavradas et al., 2014, 2016). One of these metalloproteins is a low molecular weight (6–7 kDa; 57–75 amino acids) protein named metallothionein (MT), abundant in cysteines (18–20 cysteines per molecule) with exceptional metal chelating capacity, directly involved in both toxic and essential metal detoxification and homeostasis biochemical processes (Kägi, 1991; Hauser-Davis, De Campos & Ziolli, 2012). Studies indicate suitable correlations between environmental metal levels with increased MT synthesis, making this metalloprotein an adequate biomarker concerning metal contamination (Livingstone, 1993; Lavradas et al., 2016; Hauser-Davis et al., 2017).

In this context, this study aimed to investigate the literature regarding the metallothionein (MT) mediated metal detoxification in Elasmobranchs, allowing for inferences concerning metal excretion capacity or lack thereof, and potential cellular contaminant accumulation.

## Survey methodology

The topic of metallothionein metal-detoxification in Elasmobranchs in the present study included assessments performed on any Elasmobranch species, in both laboratory and field conditions. It is important to note that papers only investigating metal loads without any MT assessments were not taken into account, as well as vice-versa.

The scientometric technique (Mota, Sampaio & Ghisi, 2019) was applied to generate qualified information on metallothionein metal-detoxification in Elasmobranchs from scientific publications indexed at Thomson Reuters’ Web of Science Core Collection (WoS), Pubmed, Scopus (Elsevier) and Google Scholar (Google) scientific databases. No grey literature was included. After manual screening of titles and abstracts and excluding duplicates and articles that did not report MT data, a total of 13 records were selected and included in the final quantitative analyses. Table 1 depicts the applied scientometric each strategy applied herein.

**Table 1:** Scientometric search strategy concerning metallothionein metal-detoxification in Elasmobranchs.

Scientometric search strategy	
Subject	Metallothionein metal-detoxification biochemistry pathway in Elasmobranchs
Scientific databases	Web of Science (Thomson Reuters), Pubmed (NCBI), Scopus (Elsevier), Google Scholar (Google)
Descriptors and boolean operators	Elasmobran* OR Shark* OR Stingray* OR Batoid* OR Guitarfish OR Chondrichthyes AND metallothionein
Language	English
Document types	Articles
Research areas	All research areas
Timespan	All years

**Note:**

Applied scientometric search strategy concerning metallothionein metal-detoxification in Elasmobranchs applied in the present study.

## Results

The applied scientometric selection resulted in very few hits, indicating that metallothionein metal-detoxification reports in Elasmobranch are extremely scarce. After the scientific database assessments, selected papers of interest on the subject were obtained (*n* = 11), displayed in Table 2. Publications ranged from 1986 to 2015, with four from the 80s, four from the 90s and five from the 2000s, all on sharks, with no data on batoids reported.

**Table 2:** Articles reporting on MT-mediated metal detoxification in Elasmobranchs worldwide.

Paper ID	Title	Year	Author
1	Cd-, Zn-, Cu-binding protein in the elasmobranch *Scyliorhinus canicula*.	1985	Hidalgo, Tort & Flos (1985)
2	Dogfish metallothionein—I. Purification and characterization and comparison with rat metallothionein	1986	Hidalgo & Flos (1986a)
3	Dogfish metallothionein—II. Electrophoretic studies and comparison with rat metallothionein.	1986	Hidalgo & Flos (1986b)
4	Effect of 2-mercaptoethanol on the electrophoretic behavior of rat and dogfish metallothionein and chromatographic evidence of a naturally occurring metallothionein polymerization.	1988	Hidalgos et al. (1988)
5	Metallothionein-like proteins in the livers of squaloid and carcharinid sharks	1990	Bonwick et al. (1990)
6	Cadmium induction of metallothionein in several dogfish organs	1991	Planas et al. (1991)
7	The trace-metal ecology of ichthyofauna in the Rockall Trough, North-Eastern Atlantic	1993	Vas et al. (1993)
8	Stage-dependent accumulation of cadmium and induction of metallothionein-like binding activity in the testis of the Dogfish shark, *Squalus acanthias*	1999	Betka & Callard (1999)
9	Antioxidant efficiency and detoxification enzymes in spotted dogfish *Scyliorhinus canicula*	2004	Gorbi et al. (2004)
10	Shark (*Scyliorhinus torazame*) metallothionein: cDNA cloning, genomic sequence, and expression analysis.	2005	Young et al. (2005)
11	Metal accumulation and metallothionein induction in the spotted dogfish *Scyliorhinus canicula*	2010	De Boeck et al. (2010)
12	Metal concentrations and metallothionein-like protein levels in deep-sea fishes captured near hydrothermal vents in the Mid-Atlantic Ridge off Azores	2010	Company et al. (2010)
13	Evaluation of the use of metallothionein as a biomarker for detecting physiological responses to mercury exposure in the bonnethead, *Sphyrna tiburo*	2014	Walker et al. (2014)

**Note:**

Articles reporting on MT-mediated metal detoxification in Elasmobranchs in the scientific literature worldwide.

**Figure 1:**
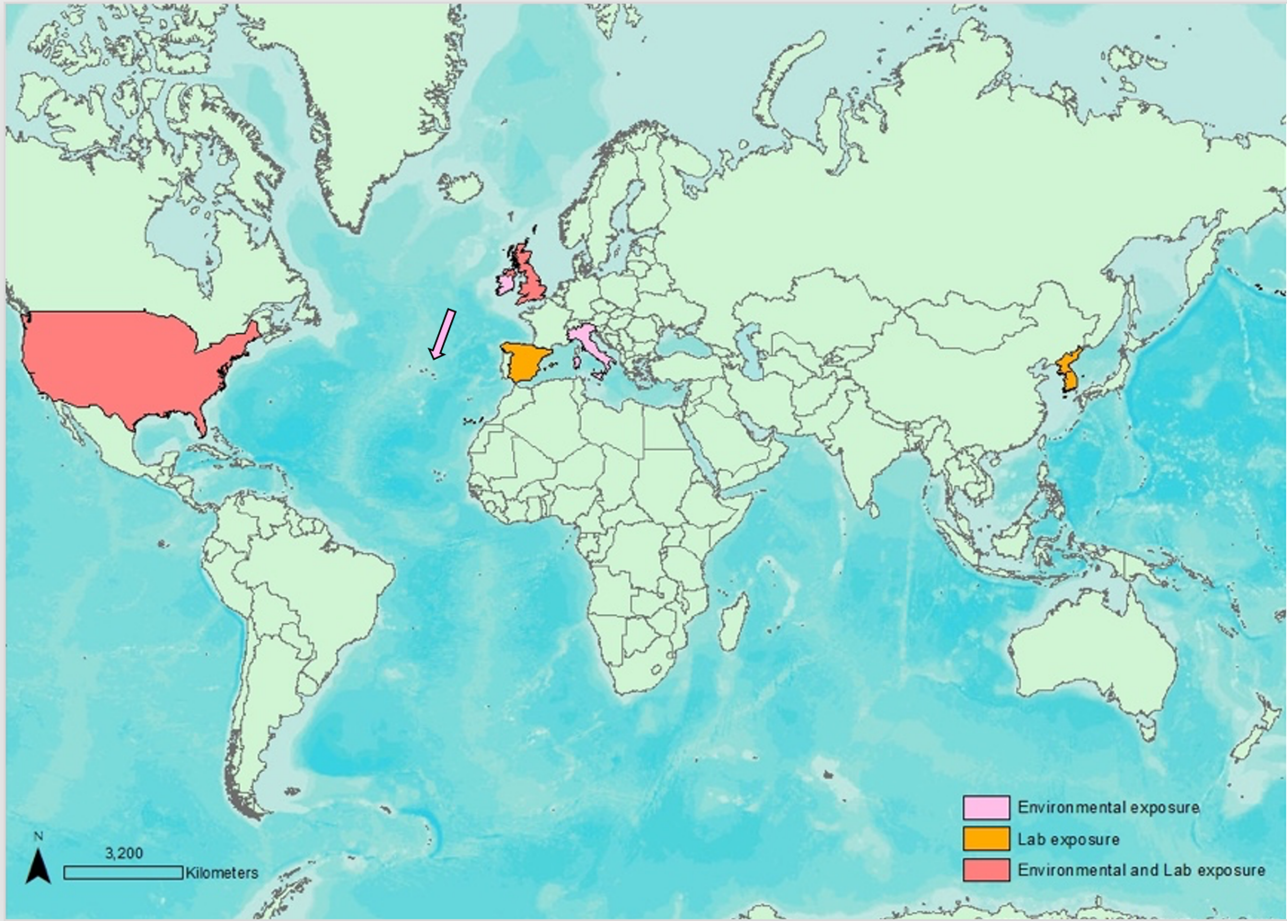
Map displaying environmental and lab exposure studies worldwide regarding Elasmobranch MT evaluations.

Figure 1 indicates the countries and type of assessment, either environmental or following laboratory exposure, or both. In this regard, only five studies evaluated MT levels in free-ranging sharks (Ireland/UK, Florida, Italy, Azores), while the others performed in vivo laboratory exposures.

Concerning investigated species and evaluated organs, a total of 15 species were reported (Table 3), with *Scyliorhinus canicula* the most representative, predominantly in laboratory exposure studies, and the most frequently assessed organ was the liver, in 10 of the 11 studies, whereas muscle and kidneys were assessed only in two studies, and others tissues, only in one.

**Table 3:** Investigated species, organs and type of evaluation reporting on MT-mediated metal detoxification in Elasmobranchs.

Paper ID	Species	Organ	Type of evaluation
1	*Scyliorhinus canicula*	Liver	Lab exposure
2	*Scyliorhinus canicula*	Liver	Lab exposure
3	*Scyliorhinus canicula*	Liver	Lab exposure
4	*Scyliorhinus canicula*	Liver	Lab exposure
5	*Etmopterus spinax, Galeorhinus galeus, Scymnorhinus licha*	Liver	Environmental analyses
6	*Scyliorhinus canicula*	Spleen, pancreas, kidney and gonads	Lab exposure
7	*Dalatias licha, Etmopterus spinax, Deania calceus,Centroscymnus crepidater, Centroscyllium fabricii, Centroscymnus coelolepis, Etmopterus princeps, Galeus melastomus, Galeus murinus, Apristurus spp*.	Liver	Environmental exposure
8	*Squalus acanthias*	Testis, plasma	Lab exposure
9	*Scyliorhinus canicula*	Liver	Environmental exposure
10	*Scyliorhinus torazame*	Liver and kidney/liver/liver and kidney	Purchased from a local market/Lab dosed with metals
11	*Scyliorhinus canicula*	Blood, gill, liver, kidney, rectal gland, intestine, muscle and skin tissue	Lab exposure
12	*Deania hystricosa, Etmopterus princeps*	Gills, liver and muscle	Environmental analyses
13	*Sphyrna tiburo*	Liver and Muscle	Environmental exposure

**Note:**

Investigated species, organs and type of evaluation in the scientometrically selected articles reporting on MT-mediated metal detoxification in Elasmobranchs.

## Discussion

The low number of articles found on the subject of the metallothionein metal-detoxification in Elasmobranchs, therefore, corroborates the significant knowledge gap in this regard, reiterated by the present assessment.

The following assessments were found dealing with the subject of the metallothionein metal-detoxification in Elasmobranch, as follows:

Articles 1, 2, 3 and 4 (Hidalgo, Tort & Flos, 1985; Hidalgo & Flos, 1986a, 1986b; Hidalgos et al., 1988) were published by the same research group. The authors isolated MT from both control and cadmium-treated spotted dogfish (*Scyliorhinus cunicula*) liver, assessed inter-sex differences and characterized MT behaviour through polyacrylamide gels, comparing shark MT with mammal MT and observing several similarities.

Article ID 5 (Bonwick et al., 1990) isolated MT from the liver extracts of three sharks, the velvet belly lantern shark (*Etmopterus spinax*), school shark (*Galeorhinus galeus*) and seal shark (*Scymnorhinus licha*), marking the first data on MT in natural populations of sharks and in mid- to deep-water species.

Article ID 6 (1991) (Planas et al., 1991) evaluated spotted dogfish (*Scyliorhinus cunicula*) specimens after exposure to to 50 mg L^−1^ Cd for 4 days and assessed MT levels in the spleen, pancreas, kidney and gonads, reporting a Cd-binding protein similar to dogfish liver metallothionein in pancreas and kidney, while postulating the existence of an analogue protein at very low concentrations in the other assessed organs.

Article ID 7 (Vas et al., 1993) determined Cu, Mn and Ni concentrations in tissue samples from prey fish and sharks from the Rockall Trough and noted differential tissue accumulation, no apparent bioaccumulation in sharks. MT were detected in liver samples from nine deep-water shark species (kitefin shark—*Dalatias licha*, seal shark—*Etmopterus spinax*, great lantern shark*—E. princeps*, birdbeak dogfish—*Deania calceus*, longnose velvet dogfish—*Centroscymnus crepidater*, Portuguese dogfish—*C. coelolepis*, black dogfish—*Centroscyllium fabricii*, blackmouth catshark—*Galeus melastomus*, mouse catshark—*G. murinus* and a species of ghost catshark—*Apristurus spp*.), at similar concentrations for all species and were independent of hepatic Cu and Cd. The authors suggest MT involvement in the homeostasis of essential trace metals.

Article ID 8 (Betka & Callard, 1999) used the spiny dogfish (*Squalus acanthias*) as a testis model to assess intratesticular Cd accumulation and effects after a single Cd injection, as this metal is an established spermatotoxicant. Cd was rapidly eliminated from plasma but accumulated and remained in testis for at least 7 days. The tissue:plasma Cd ratis in the germinal testis zone (71:1) was similar to that in liver (87:1) but lower than in kidney (381:1), and the same intratesticular gradient of germinal zone (GZ) > premeiotic (PrM) > meiotic (M) > postmeiotic (PoM) stages were noted for both treated and untreated individuals.

Article ID 9 (Gorbi et al., 2004) investigated antioxidant system and detoxification enzyme efficiencies in spotted dogfish *Scyliorhinus canicula* in comparison to a teleost, red mullet (*Mullus barbatus*). Biotransformation cytochrome P4501A activity (expressed as Ethoxyresorufin-O-deethylase) in elasmobranchs was more than one order of magnitude lower than in the red mullets, as well as lower antioxidant enzyme activity, and a more reduced efficiency in neutralizing the hydroxyl radical. Both scavenging capability toward radical oxygen species and MT levels were comparable in both groups.

Article ID 10 (Young et al., 2005), isolated novel MT complementary DNA and genomic sequences from cloudy catsharks (*Scyliorhinus torazame*), reporting that this MT shared many conserved features with other vertebrate but also exhibited some unique characteristics. In addition, the MT messenger RNA levels in liver and kidney were significantly affected by experimental exposures to cadmium, copper, and zinc, in a general dose- and time-dependent manner, through both injected and immersion exposures

Article ID 11 (De Boeck et al., 2010) examined differences in accumulation rates and toxicity of several metals (Ni, Cd, Pb, Cu and Ag) at equimolar concentrations (10 μmol L^−1^) in the Mediterranean or spotted dogfish (*Scyliorhinus canicula*) for one week, determining total metal accumulation, metallothionein induction, and parameters related to osmoregulation. The authors reported high Ag toxicity and accumulation rates and differential accumulation of the other metals in the assessed organ (blood, gill, liver, kidney, rectal gland, intestine, muscle and skin tissue), but indicated that, among the assessed metals, only Cu was associated to induced MT synthesis in liver and gills.

Article ID 12 (Company et al., 2010) investigated the concentrations Ag, Cd, Cr, Cu, Fe, Mn and Ni metallothionein-like (MTL) proteins in several deep-sea fishes captured near hydrothermal vents in the Mid-Atlantic Ridge off Azores, including the rough longnose dogfish (*Deania hystricosa*) and the great lantern shark (*Etmopterus princeps*). The authors reported high metal concentrations in the captured fish and correlations only between gill MTL and Cd in great lantern sharks.

Article ID 13 (Walker et al., 2014) investigated the relationship between muscle Hg concentrations and muscle/hepatic MT levels in bonnethead sharks (*Sphyrna tiburo*), from three Florida estuaries. Total Hg concentrations in muscle were correlated to animal size, indicating Hg bioaccumulation, but no MT correlations to muscle Hg concentrations. The authors indicate this as either because the environmentally relevant levels of Hg exposure and uptake are below the physiological threshold for inducing effects in sharks or MT is a poor biomarker of Hg exposure in these fishes.

The first articles published in the 80s (IDs 1, 2, 3 and 4) aimed at simple characterization of low weight molecular proteins from sharks, as this metalloprotein had not yet been identified in this group of animals and performed assessments on whether it displays similarities to other MT proteins, that is, induced by metals. After this period, MT then began to be applied as a biomarker for environmental contamination in natural populations, and the advent of novel analytical methodologies over time allowed for more insightful assessments on this biomarker, such as genomics assessments and the integration of several responses in a broader manner. However, significant amounts of data can still be obtained from simple and routine laboratory techniques, such as UV-Vis spectrophotometry, which is reliable, reproducible and cheap, not requiring expensive set-ups for these assessments, and the most important issue is, in fact, simple routine standardization of purification procedures prior to MT determinations, which is still lacking (Wanick et al., 2010).

Undoubtedly, very few assessments of biological effects of environmental contaminant exposure in Elasmobranchs have been carried out to date (Fuentes-Rios et al., 2005), with a significant number of species (475, or about 30% of all Elasmobranchs) classified by the International Union for Conservation of Nature (IUCN) as data deficient (DD) (IUCN, 2016). In this regard, only 15 shark species were assessed herein, and no studies were performed on batoids. In fact, practically no studies on batoids are available, especially regarding metals and the metallothionein metal-detoxification, and a total of 253 skate and ray species are classified by the IUCN as data deficient (DD) (IUCN, 2016). Therefore, the same pattern of general neglect concerning batoid assessments compared to sharks are also noted for this topic.

It is very interesting to note that, considering historical elasmobranchs studies, sharks belonging to the *Mustelus* genus, as well as *Squalus* spp., are the most used for investigative research, and are considered the most traditional animal model. The present review, however, indicated a prevalence of studies using *Scyliorhinus canicular* in assessing metal contamination and MT responses. This species has, in fact, been used a model for diverse studies, including comparative anatomy and physiology, for over a century, and will be even more utilized in the future due to the development of large-scale transcriptomic and genomic resources (Coolen et al., 2008). Thus, it is now considered an emerging and widespread model for several shark studies, due to the following characteristics: relatively small size among sharks, abundance, easy maintenance, organ zonation for cell proliferation and differentiation analyses and the fact that its whole-genome sequence may shortly become accessible (Coolen et al., 2008), and relative ease of egg-case collection and culture in laboratory aquaria (Rasch et al., 2016).

A lack of differential organ evaluations is also noted, with liver as the most frequently investigated organ, probably due to the fact that MT are known to be highly expressed in this organ, which is the main detoxifying organ of the body (Langston et al., 2002; Ploetz, Fitts & Rice, 2007; Hauser-Davis et al., 2012). In this regard, it is noteworthy that no studies have been carried out in the brain, which could be interesting regarding neurotoxic metals such as Hg and Pb (Merly et al., 2019). In addition, gonad assessments are paramount concerning deleterious reproductive metal effects (Lopes et al., 2019; Hauser-Davis et al., 2020), as several metals have been reported as causing negative reproduction effects in fish, that is, altering sperm motility, and decreasing hormone secretions (Popek et al., 2006; Ebrahimi & Taherianfard, 2011), but were observed in only one assessment herein, on male testes. Muscle assessments are also vital, not only to indicate bioaccumulation and biomagnification processes (Govind, 2014), but also due to the fact that reproductive effects have been reported above certain toxic thresholds for metals in muscle tissue (Sandheinrich & Wiener, 2011).

In addition, no evaluations concerning basic MT-influencing factors, such as seasonality (Petes, Menge & Harris, 2008; Hauser-Davis & Lavradas, 2018; Hauser-Davis et al., 2019), sexual maturity, ontogeny, reproductive state (pregnant, oocyte maturation) (Hylland, Haux & Hogstrand, 1992), reproduction mode (i.e., oviparous, viviparous) or sexual differences were carried out. Moreover, the selected studies were carried out with both the southern and northern hemisphere species, from tropical and temperate areas, but no comparisons were made for this parameter. As differences in sensitivities for several pollutants have been reported between species from different climactic zones which may, in turn, result in differential biomarkers responses (Kwok et al., 2007), this is also a knowledge gap concerning MT in Elasmobranchs that requires further investigation. Furthermore, future assessments should also include evaluations on the differences between coastal and oceanic species, as well as between benthic and pelagic species, and evaluate potential associations concerning MT detoxification and potential protective mechanisms with regard to maternal metal transfer to embryos in elasmobranchs (Lyons & Lowe, 2013; Hauser-Davis et al., 2020), among others.

## Conclusions

It is clear that data is very fragmented and notoriously lacking regarding the MT metal-detoxification in sharks, especially in environmental contamination scenarios, and no data is available for batoids. Although this hinders further discussions and insights into the ecological and physiological implications of this condition it also reiterates the need for further investigations. These are, in fact, paramount, as (i) metal pollution has greatly increased in the last decades, (ii) Elasmobranchs are highly threatened with extinction, (iii) scarce information for this group is available on which metals bind to MT for subsequent excretion in environmental contamination scenarios, and (iv) no indications of minimal MT induction thresholds in environmental contamination scenarios for this group are available.

## Additional Information and Declarations

### Data Availability

The following information was supplied regarding data availability: This is a review article, no raw data was collected.
